# Practices of care among people who buy, use, and sell drugs in community settings

**DOI:** 10.1186/s12954-020-00372-5

**Published:** 2020-05-07

**Authors:** Gillian Kolla, Carol Strike

**Affiliations:** Dalla Lana School of Public Health, University of Toronto, 155 College St., Toronto, ON M5T 3 M7 Canada

**Keywords:** Drug dealing, Drug selling, Harm reduction, Overdose, Practices of care

## Abstract

**Background:**

Popular perception of people who sell drugs is negative, with drug selling framed as predatory and morally reprehensible. In contrast, people who use drugs (PWUD) often describe positive perceptions of the people who sell them drugs. The “Satellite Sites” program in Toronto, Canada, provides harm reduction services in the community spaces where people gather to buy, use, and sell drugs. This program hires PWUD—who may move into and out of drug selling—as harm reduction workers. In this paper, we examine the integration of people who sell drugs directly into harm reduction service provision, and their practices of care with other PWUD in their community.

**Methods:**

Data collection included participant observation within the Satellite Sites over a 7-month period in 2016–2017, complemented by 20 semi-structured interviews with Satellite Site workers, clients, and program supervisors. Thematic analysis was used to examine practices of care emerging from the activities of Satellite Site workers, including those circulating around drug selling and sharing behaviors.

**Results:**

Satellite Site workers engage in a variety of practices of care with PWUD accessing their sites. Distribution of harm reduction equipment is more easily visible as a practice of care because it conforms to normative framings of care. Criminalization, coupled with negative framings of drug selling as predatory, contributes to the difficultly in examining acts of mutual aid and care that surround drug selling as practices of care. By taking seriously the importance for PWUD of procuring good quality drugs, a wider variety of practices of care are made visible. These additional practices of care include assistance in buying drugs, information on drug potency, and refusal to sell drugs that are perceived to be too strong.

**Conclusion:**

Our results suggest a potential for harm reduction programs to incorporate some people who sell drugs into programming. Taking practices of care seriously may remove some barriers to integration of people who sell drugs into harm reduction programming, and assist in the development of more pertinent interventions that understand the key role of drug buying and selling within the lives of PWUD.

## Background

Research examining drug selling (frequently referred to as “drug dealing”) has found that it is a common income generation strategy among people who inject drugs, particularly among people who report daily drug use [1, 2]. In fact, the category of “drug seller” or “drug dealer” itself is somewhat fluid, with many people who use drugs (PWUD) moving into or out of low-level drug selling depending on circumstance and economic necessity [3–5]. When examining the relationship between PWUD and people who sell drugs, PWUD often describe high levels of trust when buying from their “regular” drug seller [6, 7], and consider a long and trusted relationship with the person selling them drugs as a source of protection against overdose [8–10]. PWUD frequently cite buying from a trusted or known drug seller as a harm reduction strategy that they engage in [8–11]; despite this, harm reduction programming and research has been slow to engage with people who sell drugs directly.

With few exceptions, harm reduction programs have focused on their clients as consumers of drugs, and little attention has been paid to the ways in which people who engage in drug selling—often the very same people—could be integrated into harm reduction efforts. In the context of the North American overdose crisis, multiple interventions have been scaled up including overdose education and naloxone distribution programs, overdose prevention and supervised consumption sites, and drug checking programs [12–15]. While there has been some interest in how people who sell drugs might be integrated into drug checking interventions as part of the response to the overdose crisis [6], the integration of people who sell drugs into other areas of harm reduction programming remains underexplored, and may hold promise as a way to address continuing high overdose death rates.

In this paper, we present and analyze qualitative data from interviews and ethnographic observations of the Satellite Site program—a low-threshold, peer-led harm reduction program operating within community spaces—frequently apartments—where people gather to buy, use, and sell drugs. The Satellite Site program hires PWUD to be Satellite Site workers (SSW), who are chosen because they are well-known in their communities, and want to work as a type of community-based harm reduction worker. To do so, they are trained to offer harm reduction services to other PWUD in their social networks, and work primarily from their own homes. The Satellite Sites become community access points for harm reduction equipment and information, overdose education, and naloxone distribution, and, in some sites, monitoring of drug consumption [16, 17]. One of the unique elements of the Satellite Site program is that it works directly with people who may move into and out of drug selling; the result is that some of the SSWs are people who sell, or allow drugs to be sold within their sites. The Satellite Site program is an example of a safer environment intervention [18, 19], where people who use and sometimes sell drugs are employed to deliver harm reduction equipment and education to their peers. The program aims to improve the health of PWUD by altering the socio-spatial relations within these closed spaces in the community where people gather to use drugs. In this paper, we focus on the practices of mutual aid and support—or practices of care—that were observed within the Satellite Sites. These practices of care circulate in the harm reduction work that occurs between SSWs and their clients, and were also observed during instances of drug selling and sharing. The existence of practices of care surrounding drug selling troubles popular conceptions of drug selling as always or solely predatory or deviant [20–22], and offers insights into how people who sell drugs might be integrated into harm reduction programs more broadly.

### Drug selling and harm reduction

Focusing on drug selling within harm reduction programming is not a new idea. A key early example of this is provided by Grove [23], who argued that “real harm reduction” must focus on the key concerns of PWUD, which frequently revolve around availability of drugs, how to find enough money to buy good-quality drugs, and how to avoid detection by the police. “Real harm reduction” focuses on the harms caused to PWUD by drug prohibition, particularly the harms stemming from an unregulated drug market with no quality control or method of verifying the potency and composition of drugs, and where the risk of arrest and incarceration is ever present. These are major problems for PWUD, and Grove critiqued early public health interventions for completely ignoring them while also attempting to justify harm reduction by focusing solely on its ability to prevent HIV transmission among PWUD [23]. Highlighting that “drug use is profoundly normal”, Grove traces how prohibition and criminalization not only fail to dissuade drug use, but are also social policies that accentuate the harms associated with drug use [23]. He also highlights the drug user organizing that led to “shooting galleries” being early spaces of harm reduction, where PWUD are able to use drugs off the street and where they do not have to hurry to attempt to avoid law enforcement [23].

While many key components of public health interventions for PWUD (such as needle and syringe distribution programs) started out as survival strategies among PWUD, their uptake and operationalization by public health authorities (who may have little to no connection with communities of PWUD) can lead to services that are not accommodating nor reflective of the needs of people who use drug [24]. A prime example of this is the way in which public health interventions have almost completely ignored drug buying and selling except to prohibit it within formal programs and service offerings. Due to criminalization, drug selling represents a particularly contentious issue to be managed for organizations providing services to PWUD, necessitating an institutional focus on rules and regulations. This includes rules that prohibit the sharing or selling of drugs, or—within the context of supervised consumption services—that specify particular methods of drug administration and whether people can receive assistance with injections [25, 26]. This focus on the enforcement of rules and regulations can alienate and exclude PWUD from accessing much-needed harm reduction services [25, 27, 28]. Unsanctioned and peer-run services that are designed to be “low-threshold” and function with fewer rules (or with behavioral guidelines that emerge organically from communities of PWUD) may attract more marginalized service users who would otherwise choose not to access or be excluded from accessing services [25, 26, 28–30].

The difficulties in ensuring that the institutional rules and regulations that surround the delivery of harm reduction services are low threshold and reflect the local culture among PWUD are amplified in any attempt to work with people who sell drugs, despite the frequent acknowledgment that engaging in low-level drug selling is common for people with high frequency or daily drug use [1, 2]. Alternate framings of drug selling remain rare. Research has documented how PWUD frequently engage in “helping” and mutual aid behaviors—such as procuring and sharing drugs with each other—that are technically drug distribution or trafficking offences under drug laws [31, 32]. Previous research has also documented how the mutual aid that emerges from social ties between PWUD can be protective in cases where people cannot secure an adequate supply of drugs for themselves, and must rely on other PWUD to supply them with opioids as they attempt to avoid opioid withdrawal [33, 34]. Programs that formally and explicitly engage with people who sell, share, or exchange drugs (activities that are frequently and often interchangeably referred to as “drug dealing”) in pursuit of harm reduction or public health goals are rarely documented. Framings of people who sell or supply drugs as predatory and morally reprehensible remain popular and enduring [20, 35]. This renders it difficult to explore the practices of mutual aid and support—or practices of care—that surround drug selling and sharing in communities of PWUD.

### Practices of care in harm reduction

This paper explores the practices of care that SSWs engage in as part of their harm reduction work, with a focus on the practices of care that circulate around drug selling. Drawing on Foucault’s ethics as well as more recent work on pleasure in drug use [36], Duff examines how “drug users cultivate and sustain practices of care in contexts of social and economic disadvantage” [37], (p83). Here, “practices of care” refers both to the practices used by PWUD to care for the self within larger social and economic contexts that remain hostile to drug use, as well as the ways in which people care for others, emphasizing the often relational character of practices of care [37]. Research on the practices of care surrounding drug use has emerged from a concern that the experience of pleasure has been neglected in research on illicit drug use [38, 39]. Instead, a focus on the risks and harms associated with drug use has reinforced the “pathologizing tendencies” of research on drug use, with drug policy and programming reflecting this view of drug use as almost exclusively harmful [36, 40]. In contrast, “thinking with” pleasure in drug use may provide a way to counter this tendency towards pathologization, allowing for examinations of the pragmatic ways people use drugs to manage illness or pain, and to experience pleasurable sensations and states of consciousness, in addition to the oft-documented negative effects of drug use [38, 41]. Nuancing drug use in this way may hold potential to counter stigma and moral judgment against PWUD, and open space to explore how practices of care circulate among PWUD [37].

Much of the concern with care stems from the work of feminist scholars in science and technology studies, and stems from the feminist concern with devalued labor [42]. Here, the concept of “care” carries multiple and often interwoven meanings, including care as indicative of paying attention to something in a careful or watchful manner, “caring about” as a state of being emotionally attached to something, and “caring for” as a way of providing for or looking after someone as part of a social relationship [43–46]. We focus on “practices of care” to reflect care as an active practice, a moment of active “doing” of care that engages practitioners in their worlds [42, 43]. However, it is also important to note that care is a contested concept, and that practices of care are not neutral or uniformly positive acts of affection or attachment [46]. Instead, the concept of care is constrained by uneven and asymmetrical power relations that dictate which practices of care are worthy of attention and to “count” as care [46]. Practices of care are often embroiled in a complex politics that determines which practices, people, and phenomena are recognized as caring or worthy of care and which are excluded from recognition or analysis [43]. Acknowledging this contested “politics of care” highlights how attention to care is selective, where some lives and phenomena are included and attended to, while others are neglected and excluded from consideration or analysis—particularly those marginalized by drug use and related social inequities [43, 47]. Focusing on “real harm reduction” [23] by listening to the expressed needs of PWUD around the centrality of drug buying in their lives, and developing interventions that take seriously the practices of care among people who sell drugs, provides recognition of the practices of care among a marginalized group of people that are frequently ignored.

Practices of care in harm reduction proliferate, yet have only recently begun to be described as such. Examining the practices of care among PWUD builds off research that has repeatedly documented the mutual aid among PWUD. This includes the ways that PWUD (whether formally employed as “peer workers” or not) support others within harm reduction programs and overdose prevention sites, with more recent research exploring their experiences reversing overdoses in housing and community settings [17, 47–51]. Practices of care range from the provision of sterile injection equipment to the administration of naloxone to reverse a life-threatening opioid overdose. For example, Fraser describes the “ethos of community care” that inspires people to maintain large supplies of sterile injection equipment on hand so that they can engage in secondary distribution of this equipment—a practice of care—to people in need [52]. Similarly, the way in which the expansion of take-home naloxone programs has allowed new practices of care to develop between a person administering naloxone and a person who is overdosing, has been explored to describe how people will use naloxone to gently reverse overdose, in an attempt to avoid the harms of precipitated withdrawal from more forceful naloxone administration [47].

Increased attention is being paid to how a strong focus on risk and harm in research on drug use may obscure the wide variety of drug use experiences [37, 38, 40]. However, such nuance is lacking when examining drug selling. People who sell drugs are routinely framed not only as uncaring, but as actively predatory towards others [20–22]. Many studies of drug selling and drug markets focus on the aggression, theft, and violence that can occur around drug selling and buying within unregulated drug markets [3, 53, 54]. Without ignoring the existence of these important issues, we attempt to broaden the discussion of what constitutes “care” within harm reduction practice by exploring the variety of practices of care that people are engaging in related to drug selling and drug sharing. Starting with Grove’s insight that “real harm reduction” must attend to the primacy of drug buying and selling in the lives of PWUD [23], in this paper we explore the practices of care that surround drug procurement, buying, and selling. Exploring the practices of care that exist among people who use and sell drugs opens the possibility for a reconceptualization of some aspects of drug selling more generally, and for imagining a place for the broader participation of people who sell drugs in harm reduction programs specifically.

## Methods

### Setting

This research was conducted with the “Satellite Site” program, implemented in Toronto, Canada. The Satellite Site program started informally in 1999, as an outgrowth of a peer-developed and peer-run harm reduction program operating inside a community health center. The founder of the program, who ran the harm reduction program at the community health center and who openly identified as a person who injected drugs, began to provide home delivery of sterile injection equipment to increase access for community members outside the hours of operation at the community health center. Noticing that many people maintained communal spaces where people gathered to use illicit drugs, he began asking the people running these spaces if they wanted to keep extra harm reduction supplies around for others who might need them; this was the beginning of the Satellite Site program [16]. The Satellite Sites were based on a secondary syringe exchange model, where PWUD would distribute sterile injection equipment obtained from formal harm reduction programs to other people who inject drugs out in the community [55–57]. Since he was familiar with the sites and had spent time observing their operation firsthand, the program founder was able to choose Satellite Sites deliberately, and was able to assess potential Satellite Site workers for their suitability and privilege choosing spaces that were already engaged in high-volume needle and syringe distribution and disposal. Due to his close connections with the community, the program founder was also able to verify that the people running the sites were interested in working within a harm reduction philosophy and were not formally associated with any criminal organizations. He also ensured that SSWs were well-connected to the community health center so that they could provide referrals to the center for healthcare and social service needs. In 2010, the Satellite Site program adopted a more formal model when the program received external funding [16]. This allowed for the SSWs—who were previously in a volunteer role—to be employed as paid staff, receiving a modest salary of $250 a month and cellular telephone. During the study period, there were 9 Satellite Sites in operation, and each distributed and disposed of, on average, approximately 1500 needles and syringes a month.

### Data collection

A community-based research approach was used to guide the data collection in this ethnographic study. The lead author was well-known to SSWs due to previous involvement in an evaluation of the program [16], as well as work on other research projects in the community health center. An advisory group consisting of key members of the program, including the program founder, the Satellite Site coordinator, and several SSWs met regularly with the lead author to provide guidance on research design, data collection and recruitment, and on the interpretation of the data. Research ethics board approval was obtained from the researchers’ institution.

SSWs were invited by the lead author to participate in site visits and/or one-on-one semi-structured interviews. SSWs who expressed interest in participating provided informed consent for observations at their site prior to the first visit. The consent process included a discussion of how they could opt out of site visits, ask the researcher to leave at any time, or withdraw from the study completely. As part of the consent process, SSWs were asked to inform any Satellite Site clients who were at the site at the time of a visit about the research study using a short script. Ethnographic observation at the Satellite Sites occurred over a period of 7 months, from September 2016 to March 2017. In total, 57 observation visits were conducted. Sampling was guided by the sequential approach described by Small [58]. Visits occurred once or twice a week, and averaged an hour (with visits ranging from 15 min to 2 h in length). They generally occurred in the evenings, with visits held on different days to capture variations in operations between weeknights and weekends. Days and times of observation visits were arranged in advance by the Satellite Site coordinator, an employee of the community health center responsible for supervision of the Satellite Site program who regularly visited the sites as part of their job responsibilities and who was present during all site visits. SSWs were provided a $20CDN honorarium at each visit. Field notes on observations were recorded immediately after leaving sites, and expanded upon in detail the following day, using principles outlined by Emerson et al. [59]. An observation guide was used to highlight major areas of attention, and focused on instances of drug use observed (including the buying, selling, preparation, and consumption of drugs), any instances of violence or aggression, interventions by police or paramedics, and interactions between SSWs and clients where harm reduction interventions occurred, including the following: equipment distribution, harm reduction education, naloxone distribution, overdose education, overdose intervention, and provision of information and referrals to health or social services. All field notes were anonymized using pseudonyms, with names of participants and locations of Satellite Sites never recorded in field notes due to the criminalized nature of activities being observed.

Seven Satellite Sites were visited on a regular basis. Two of these Satellite Sites were in privately owned apartment buildings, and five were located in subsidized social housing complexes. The closest Satellite Site was located 2 km from the sponsoring community health center, and the farthest was located 11 km away. The SSWs running these seven sites included six individuals and one couple; three men and five women, all between the ages of 45 and 70 years old who injected drugs regularly (although their drugs of choice varied), and who received much of their income from government social assistance programs.

One-on-one, semi-structured interviews were also conducted to complement the information gathered during observation visits. SSWs were interviewed twice; once prior to the beginning of observation visits in the Satellite Sites, and once following the completion of visits. In the first interview, they were asked about general issues relating to their work as a SSW, such as how they became part of the program, challenges they faced, and benefits of being a SSW. The second interview was used to expand on issues and themes that emerged from the observation visits. Clients and supervisory staff were each interviewed once only. Clients were recruited from the Satellite Sites, where the researcher would discretely approach them to determine interest in participating in a confidential interview in a spare room in the Satellite Site (if available), or at another location (e.g., a coffee shop or community health center). They were asked about the experience of visiting the Satellite Site, and their use of other health or harm reduction services. Community health center staff were also interviewed; staff were all involved in different aspects of administering or supervising the Satellite Site program. They were asked about institutional factors associated with running the program, such as challenges faced in implementation or program expansion. All interview participants gave verbal informed consent to be interviewed, and were offered $20CAD honorarium.

In total, 15 participants were interviewed, including five SSWs, four Satellite Site clients, and six staff members from the community health center who administered and worked in supervisory roles over the program. Two of the SSWs who were interviewed were men, and three were women, and all were between the ages of 51 and 70 years old. They all injected drugs regularly, with four injecting daily, and four lived in subsidized housing complexes, with the remaining SSWs living in a privately owned apartment building. For Satellite Site clients who participated in formal interviews, three out of four were male, between 20 and 30 years of age, with the fourth participant in the 50–60-year age range. One client was homeless, with the remaining three living in subsidized community housing.

### Data analysis

Data analysis was guided by a theoretical approach that aimed to foreground the ways in which a marginalized group of people engage in harm reduction work in community settings, with a focus on how structural and social forces shape actions that are often viewed (and framed) as individual-level risk behaviors [60–62]. This approach shaped the focus on care as a practice, and on the ways that practices of care were being actively enacted by SSWs in their work with PWUD in the Satellite Sites. Field notes and interview data were analyzed using an iterative process guided by thematic analysis [63]. Beginning in the early stages of participant observation, field notes were coded for key themes. As data collection progressed and interviews were conducted, emergent themes were grouped by category. The ways in which SSWs engaged in thoughtful practices of care within their work was identified in subsequent iterations of coding as a major analytic trajectory. Later iterations of data analysis refined this analysis by focusing on the interactions between drug selling and acts of care. Dedoose qualitative data analysis software was used for data management and coding. In the excerpts below, all participants are identified by pseudonyms only, and potentially identifying details have been altered.

## Results

### Drug selling and practices of care

The Satellite Site program integrates the distribution of harm reduction materials directly into the spaces in the community where people are already gathering to buy and use drugs, and helps make sterile drug injection and unused inhalation equipment more widely available in these community settings. During the observations for this study, people were repeatedly observed visiting Satellite Sites to get harm reduction supplies and to make use of the sterile equipment available. No instances of needle or syringe sharing were noted. Clients of the sites recognized the health-related benefits of the widespread availability of sterile injection equipment at the Satellite Sites. As one client indicated: “It's probably allowed me to avoid certain health complications. Like, I don't have Hep C, right? I don't have HIV or anything like that. So that's a positive” (interview with Satellite Site client 1). This client also stated that he had never accessed a formal harm reduction program, and accessed all his injection equipment at a Satellite Site where drugs were being sold. This speaks to the long history of secondary distribution within harm reduction, where PWUD maintain a reserve of harm reduction materials to distribute to people who may not otherwise be accessing formal harm reduction programs [55, 64, 65]. Secondary distribution has been well-documented even in jurisdictions where it is illegal, as continues to be the case in Australia [66]. The distribution of harm reduction materials—including the stockpiling of large amounts of equipment by PWUD for friends, family, and community members to use—has been described as a practice of care for others [52]. The Satellite Site program formalizes this practice of care to further the public health goal of improving access to sterile injection equipment, as this is a key public health intervention to reduce the transmission of bloodborne infections [67].

Satellite Sites are not required to allow either drug use or drug selling within their sites: SSWs decide for themselves how they wish to run their sites. The Satellite Site program goes beyond the traditional public health focus on the distribution of harm reduction equipment and moves towards “real harm reduction” by acknowledging the ways that buying, using, and sometimes selling drugs are intertwined within the lives of PWUD [23]. Unlike traditional harm reduction programs located in community organizations or health centers where drug use and drug buying, selling, and sharing are not allowed, the Satellite Site program is designed to be located in places in the community where these activities are already taking place. The program recognizes that while access to sterile equipment for drug use is important for PWUD, it is not necessarily their top priority. The following field note is from a Satellite Site run by Phil, a man in his 50’s who injects heroin and also engages in small-scale heroin selling to friends and acquaintances who live in his apartment building. It illustrates the benefits of co-locating harm reduction equipment within the spaces where people are buying and using their drugs:

There is a knock at the door, and Phil walks over, opening the door a crack. He speaks quietly for a moment to the person at the door, before letting a woman in. Phil asks her what she needs, and she quietly says, “Can I get some kits, and a point *[of heroin]* too?” He goes into the kitchen, grabs an empty grey plastic bag, and passes it to her, gesturing to the stack of plastic drawers holding all the harm reduction supplies. She opens the top drawer, takes a bunch of injection kits (that contain sterile needles, cookers, filters, water, and tourniquets), and then grabs a handful of crack pipes, putting them all in the bag. Phil heads to the bed, and, sitting on the edge, he pulls out a scale and a tiny Ziploc bag. The woman sits on the floor in front of him, watching intently as Phil shakes a little bit of heroin powder onto a small square of paper that he’s placed on the scale. He’s going slow, tipping powder out of the baggie, carefully weighing the drugs out. Once he gets to the right number on the scale, he looks up at her, and she nods, before he carefully folds up the little piece of paper. She passes him some money, says thanks, and nods a goodbye at us. (Field note 2016-11-19)

Increasing access to sterile injection equipment within the spaces where people are buying and using their drugs can facilitate the important public health goal of preventing transmission of HIV and hepatitis C. Additionally, combining the provision of sterile injection equipment with drug purchasing can enable small practices of care in the lives of people who may not be accustomed to receiving care around their drug use.

Another example of a practice of care occurs when SSWs use their personal knowledge of drug potency and translate it into harm reduction education. Adrienne is a long-time harm reduction worker in her 50’s, and runs a Satellite Site out of her apartment. Her clients are mostly friends and family members, and she knows their drug use habits very well. She injects opioids but does not sell them, and mobilizes her personal experience to attempt to prevent overdose among clients:

They *[client]* will say, 'Oh, what's the junk like?' And I'll say 'I don't know. Which one did you get?' and they’ll show me: 'This one’. And I can tell them, ‘Be careful, it's a little strong. You can always do more'. But also, they know, ‘Don't worry’, you know, ‘I'll keep an eye on you’. (Interview with SSW 8)

Here, Adrienne references both her knowledge of drug potency and her ability to intervene in case an overdose occurs. The Satellite Site program formalizes overdose response as a practice of care by providing SSWs with training in overdose intervention and equipping the sites with naloxone kits that could be used onsite or distributed to their clients [17]. For clients who use the Satellite Sites, the combination of harm reduction services and quick intervention in case of overdose created a feeling of safety:

I like that it's a place that's safe and you know nothing's going to happen to you. And shit, Adrienne *[the SSW]* has all the supplies and everything. For example, like, when people overdose, she has everything ready. And she’s actually used it *[naloxone].* (Interview with Satellite Site client 2)

The Satellite Sites were developed as a way of building on the practices of care that exist between PWUD surrounding the distribution of sterile needles and syringes. In the context of the overdose crisis, SSWs also mobilize practices of care to improve overdose prevention and response in the community.

### Drug potency, overdose, and practices of care

Over the period of field work for this study, the contamination of the illegal heroin supply with illicitly produced fentanyl and fentanyl analogs translated into an increase in the frequency of overdoses within Canada; this increase in overdose was also seen within the Satellite Sites [17, 68]. There are strong indications that fentanyl and its analogs are entering the drug supply chain early, likely in source countries [69, 70]. Those involved in street-level drug selling are low on the supply chain and often unaware of the content or potency of the drugs they are selling. The following field notes describe how Tommy—a SSW who also sells heroin—engages in a practice of care related to overdose prevention:

I arrive at Tommy’s place with Sonia, who is looking to buy some heroin from him. After knocking for almost 10 minutes, Tommy finally answers the door. He is clearly very intoxicated, sedated. His speech is disconnected, and he is walking slowly around his apartment running a hand through his hair, barely able to keep his eyes open. “I think I was totally unconscious. I did some dope, Sam brought some new dope over, and it was crazy. You know me, I’m no lightweight, but it just knocked me out. And I only had half a point! That stuff is crazy strong.”

Hearing this story, Sonia pipes up and says, “Hey, Tommy, could I get a bit of that from you? I’ve got 40 bucks. I’ll take some of that good stuff that knocked you out. I can take that off your hands, if you want.” Tommy shakes his head, and says, “No way! There’s no way I’m selling that – it’s too strong. Maybe it’s cut with fentanyl, but it doesn’t feel heavy like fentanyl – it had the smooth, gradual start like good junk. But it’s really too strong. That could kill someone, and I don’t want to be responsible for that.” He takes a dime bag out of his pocket, and takes out a small chunk of beige heroin, putting it on the table. It looks a bit like silly putty, and he takes a kitchen knife from the table and cuts a small chunk off the end, saying, “But don’t worry, this stuff is good too.” (Field note, 2016-12-14)

This field note complicates the simplistic narratives that portray people who sell drugs as reckless and indifferent to the wellbeing of their clients. Here, Tommy is troubled by the strength of the heroin he has just consumed, and is unwilling to sell it to Sonia, as he worries that it might cause a fatal overdose. This field note points to the potential benefits of integrating people who sell drugs into harm reduction programs within the context of the current opioid overdose crisis.

### Mutual aid, drug procurement, and practices of care

The example in the field note above is an illustration of a practice of care in relation to drug selling. However, the heavy stigma surrounding drug selling can make it difficult to recognize this and other common practices of care that circulate around drug buying and selling. Common, normative narratives frame people who sell or procure drugs in almost exclusively negative terms, as reckless, predatory, and unconcerned about their clients [20, 21]. The fieldwork for this study challenges this portrayal: PWUD were frequently observed engaging in mutual aid and practices of care to assist each other with drug procurement, which is sometimes referred to as social supply. In social supply, drug transactions within social networks are facilitated either at cost as a means of reinforcing social ties, or at a small markup to compensate the seller for their effort, risk, or to allow them to finance their own use [31, 71]. Helping other people to procure drugs is very common; however, these acts are nonetheless drug trafficking offences. SSWs would frequently engage in these types of social supply or “helping” behaviors, despite the risk of arrest. During observation visits, a form of mutual aid was frequently observed where clients and SSWs pooled their money and arranged to “pick up” from a drug seller:

SSW: And I pick up for people too, so. By me picking up, I'm usually, you know, I'm in and out, in and out, in, I'm surprised I haven't got nabbed yet, knock on wood. (laugh)

Interviewer: And what's in it for you for picking up for them?

SSW: It depends. From the dealers, I get, you know, some extra. From the people themselves, they'll throw me something. I usually go if I'm making money. (laugh) If I'm not making money, I don't want to go. (laugh) I mean, it's not a lot of money but it helps.” (Interview with SSW 7)

Pooling money for a pickup was mutually beneficial, both from an economic perspective as the people putting in money would receive a better deal, and because assisting with these transactions helped sustain and cement social relationships. There is also risk involved in providing this help due to criminalization. These helping behaviors are complex, as they comprise elements of assisting others to procure drugs and self-interest in acquiring free drugs.

Additionally, some SSWs were observed helping their clients to negotiate the vagaries of drug markets that lack formal dispute resolution procedures. For example, Sandra, a SSW who did not formally sell drugs, explains how she would often help to procure drugs for Satellite Site clients who were unable to buy drugs due to disputes or unpaid debts with drug sellers:

Service users know that I do have a good rapport with dealers, and good credit. So sometimes, that will come up, like, you know, 'Oh, well, you get it and then I'll pay you’. (Interview with SSW 10)

The Satellite Site program was built around the practices of care surrounding harm reduction equipment distribution within communities of PWUD. By recognizing the importance of drug buying and selling in the lives of PWUD, the practices of care circulating between SSWs and their clients around drug procurement can be rendered visible. Revealing the diversity of relationships and arrangements that surround drug selling may contribute to decreasing the pathologization associated with this often stigmatized practice (37, 47). The following field note from a busy Satellite Site provides an example of how social supply networks function, and how a SSW was motivated to assist in the procurement of drugs out of a desire to provide assistance to community members:

It’s Saturday night and we are at Bobby’s place, sitting in the living room with the TV on in the background. It’s been busy, with people coming in and out all night, picking up both injection kits and crack smoking kits. There is a knock on the door, and Bobby comes back with Stella. She says hello to me, as we hadn’t seen each other in a while. She turns to Bobby and somewhat sheepishly says, “Bobby, can I ask a favour? Would you mind getting some crack for me, a 40 piece? Your guy, it’s good stuff?” Tom perks up, and answers, “Oh yeah, it’s good. It’s a little grainy right now, like, it’s not big rocks, but it’s good.”

Stella, looking visibly relieved, replies, “Okay, would you mind? My guy, he usually comes to me, he delivers. But he’s not working right now. And I know someone else, but we have to meet on the street. And I hate that. I hate standing out on the street, waiting, not knowing if there are cops around. I get so nervous, I hate it. And I feel like the guy I’m seeing now, that he’s shorting me. Maybe it’s just paranoia, but it seems like the bags are getting smaller and smaller.” Stella hands $40 to Bobby, and he gets up, heading towards the door to his apartment, saying, “No problem, I’ll be right back.”

Stella and I make small talk and watch TV while we wait. About 10 minutes later, Bobby comes back, and hands her a little baggie. Stella looks at it quickly, saying, “Thanks. This is 40?” She’s looking closely at the rocks in the bag, and I can tell that she is unsure about the quantity, and probably wondering if she is getting ripped off. Bobby replies to her, “Yeah, this is good stuff. See what I mean, it’s a little grainy? But that’s the same stuff I had earlier. It’s good. Do you need pipes?” Stella, still looking at the bag, and probing it with her nail, replies, “Yeah, that would be great, thanks.” Tom packs up a bag of new crack pipes, filters, and push-sticks for her to take. (Field note 2016-11-12*)*

The importance of ensuring an adequate supply of good quality drugs, while also avoiding getting ripped off and being criminalized for drug use is highlighted as a key concern within “real harm reduction” [23]. However, it is not frequently attended to within drug policy or harm reduction research, despite the prominent role it plays in the lives of PWUD. In the field note above, Tom is making it possible for Stella to avoid a situation that was clearly causing her great stress and anxiety, by procuring drugs for her. Stella’s expressed fears of getting ripped off and of encountering police (and by extension, being criminalized for her drug use) are frequent among PWUD. Attending to the primacy of safety during drug procurement is not a traditional concern of public health-oriented harm reduction activities. This is partly due to normative framings of drug use as negative and dangerous, which can make it difficult to see acquiring, procuring, or selling drugs as a practice of care, since drug use is almost exclusively framed as dangerous and having no potential of positive benefits for the person using them [37]. Validating these acts of mutual aid as practices of care opens the possibility of integrating interventions around drug selling into harm reduction programming.

## Discussion

The findings from this ethnographic research document the practices of care occurring around drug buying, using, and selling within a community-based harm reduction program staffed by SSWs—some of whom are actively engaged in drug selling alongside their harm reduction roles. Common portrayals of PWUD, and particularly people who sell drugs, as solely predatory, lacking in self-control, or as careless obscure the practices of care that were observed in this study. These negative portrayals also make it difficult to integrate these practices of care—and the people who sell drugs who practice them—into public health programming to facilitate harm reduction goals. Harm reduction programs are well-placed to work with people who sell drugs due to their philosophy of meeting PWUD “where they’re at,” of integrating PWUD within program development and delivery, and of highlighting the health harms associated with the criminalization of drug use. Our findings suggest that there is potential to expand the relevance and reach of harm reduction programming by recognizing the practices of care that occur around drug selling, and integrating them into harm reduction. The criminalization of behaviors that we observed as common among people who use drugs (such as buying drugs together, buying for another person, and pooling money to buy and share a larger quantity of drugs) contributes to the difficulty in making visible the practices of care circulating within drug buying and selling. The criminalization of drug selling has also rendered these behaviors highly stigmatized. While the idea of integrating PWUD into harm reduction programming and service delivery in the response to the overdose crisis is not new [50], there has been little formal integration of people who sell drugs into harm reduction programming. Highlighting these practices of care has the potential to remove barriers that hinder the integration of people who use and sell drugs into harm reduction programming, and to develop public health interventions that take seriously the key role of drug buying and selling within the lives of PWUD.

Some of the practices observed in this study are easily recognizable as “care” (e.g., the provision of equipment for drug use and intervention when overdoses occur) because they align with traditional framings of affective care work as a form of looking after another, or “caring for” someone [46)]. These practices of care are already well-established and practiced within harm reduction programming, particularly in harm reduction programming that utilize “peer” workers [50, 72]. Offering harm reduction equipment in the same location as drug selling allows for more widespread dissemination of sterile injection equipment, a key public health strategy in reducing the transmission of HIV and hepatitis C. In association with drug selling, these more traditional practices of care lead to increased trust and help facilitate harm reduction education and equipment distribution.

However, other practices we identified are frequently overlooked as “care” because they are often associated with criminal acts (e.g., assistance in procuring or buying drugs, the provision of information on drug potency, or refusing to sell drugs that are deemed to be too strong). Our results underline how behaviors such as buying drugs together, buying for another person, and pooling money to buy and share a larger quantity of drugs are common within the social networks of PWUD. Despite their frequency, as well as their role in decreasing and sometimes preventing overdose, these behaviors are nonetheless heavily criminalized as drug distribution or trafficking under current drug laws. The criminalization of commonplace behaviors contributes to alienating people who use and sell drugs from the health and social service system, and results in missed opportunities to engage people who are selling drugs in potentially beneficial public health interventions [1, 54]. There is potential to expand the relevance and reach of harm reduction programming by recognizing the practices of care associated with drug buying and selling, and integrating them into formal harm reduction programming. The Satellite Site program recognizes and builds upon the practices of care that SSWs are already engaging in within their communities (such as secondary syringe distribution). The inclusion of people who sell drugs in formal roles within harm reduction programming is not only a recognition that they are already engaging in practices of care within their communities, but can contribute to shifting the possibilities of care by “contingently co-producing different capacities for, and subjects of, care” [73] (p 433). This is particularly the case in the context of the overdose crisis in North America; our finding that SSW transmit important information on drug potency to their clients highlights an important potential role within harm reduction programming for people who sell drugs in the transmission of information on drug potency within social networks.

A key feature of the Satellite Sites is that the drug selling sometimes occurring within them is not seen as a liability, but is utilized to achieve public health goals like increasing access to sterile equipment for drug use. Secondary distribution programs have long been used to increase the reach of more formal harm reduction programs [55–57]. By situating a harm reduction intervention directly in the spaces where drugs are being bought, used, and sold, the Satellite Site program operationalizes Grove’s insight that the key concerns of PWUD revolve primarily around how to find and purchase good quality drugs [23]. The program then extends this insight by training PWUD who may move into and out of drug selling as peer harm reduction workers. In doing so, the Satellite Sites provide an example of how to reduce the structural barriers faced by PWUD that impede access to health and social services by providing convenient access to sterile injection equipment directly within the environments where drugs are sold. Importantly, this program also recognizes and formalizes the practices of care surrounding secondary distribution that are already occurring within communities of PWUD [52]. Similar to calls for exploring the potential integration of people who sell drugs into drug checking interventions [6], our findings suggest that an expanded recognition of what “counts” as care to encompass the practices of care that circulate around drug selling can be mobilized within harm reduction programs in an attempt to address the overdose crisis. Provision of information on drug potency and integration of people who sell drugs into drug checking interventions represent a promising area for expansion for harm reduction programming. It acknowledges a potential role of people who sell drugs in addressing overdose risk from the wide variations in the composition of the opioid supply. Additionally, people who sell drugs represent an avenue for reaching “hidden” populations of PWUD, who are unconnected to formal harm reduction programs or other health services.

Mobilizing PWUD as peer workers within harm reduction programming and in response to the overdose crisis is an important strategy to expand service delivery and improve engagement with harm reduction services [50]. However, there is a strong need to ensure that this care work is not downloaded onto peer and community harm reduction workers in ways that may reinforce existing marginalization [17, 47]. Scholars have highlighted the potential for care work to be exploited and/or exploitative, particularly since care work is traditionally devalued, as it is frequently gendered, unpaid, and associated with groups experiencing marginalization [42, 43]. Criminalization of drug use and drug selling contributes to the marginalization of PWUD as they take on work within harm reduction, which has led to documented inequities in stipends, salaries, and access to benefits, contingent work, and lack of job protections [50, 72]. Harm reduction workers—even those who sell drugs—must be adequately compensated for their labor, and provided with proper supports to address the grief and negative emotional impacts associated with providing frontline services during the overdose crisis [17, 50].

Care must be taken when attempting to extrapolate the findings from this study to other geographical settings, as not all environments or contexts would be amenable to this type of intervention. For example, ethnographic research in Montreal, Canada, exploring “piaules” (the local term for “crack houses”) found that these spaces were closely tied to and often operated by criminal organizations, rendering the integration of harm reduction programming into them very difficult [74]. The Satellite Sites, by contrast, are run out of private apartments by individuals who are not associated with any criminal organization. As mentioned earlier, the founder of the Satellite Site program was very deliberate and took great care in the selection of the Satellite Sites. He used both his familiarity with the community settings where people gathered to use drugs and his in-depth knowledge of harm reduction practices to choose potential Satellite Sites. An in-depth examination of the local norms within the spaces where drugs are used and sold in the community, in partnership with PWUD who are knowledgeable about the local context, is necessary prior to the development of any programming that aims to integrate people who sell drugs into harm reduction service delivery.

## Conclusion

The increased prevalence of fentanyl in the illicit opioid market in most parts of North America has led to a massive increase in overdose deaths in the USA and Canada [68, 70, 75]. It also constitutes a changing risk environment for PWUD [76], where the presence of fentanyl in the illicit opioid supply is disrupting the ways in which both PWUD and people who sell drugs attempt to keep themselves and others safe from overdose [69, 70]. Available information suggests that this contamination is happening far up the supply chain, and not primarily among street or low-level drug sellers [69]. Findings from this study suggest that there is strong potential to integrate low-level drug sellers—particularly those who know their clients well and are already engaging in practices of care towards them—in harm reduction programming. PWUD have already been integrated into community based initiatives that equip them to intervene quickly when overdose occurs [17, 50, 77]. Expansion to formally engage some people who sell drugs in overdose response initiatives, transmission of information on drug potency, or in drug checking interventions [6] may provide additional avenues for capitalizing on existing practices of care among people who sell drugs, and addressing the opioid overdose crisis at the community level.

## Data Availability

The datasets generated and analyzed during the current study are not publicly available given the sensitive nature of the research topic, as they contain confidential information that could compromise participant confidentiality and consent.

## Abbreviations

PWUD: People who use drugs
SSW: Satellite Site worker
Hep C: Hepatitis C
